# Meglitinide (repaglinide) therapy in permanent neonatal diabetes mellitus: two case reports

**DOI:** 10.1186/s13256-021-03052-5

**Published:** 2021-10-25

**Authors:** Maryam Razzaghy-Azar, Mitra Nourbakhsh, Ali Talea, Mahsa Mohammad Amoli, Mona Nourbakhsh, Bagher Larijani

**Affiliations:** 1 Metabolic Disorders Research Center, Endocrinology and Metabolism Molecular-Cellular Sciences Institute, Tehran University of Medical Sciences, Tehran, Iran; 2 Pediatrics and Pediatric Endocrinology and Metabolism, Hazrat Aliasghar Children’s Hospital, Iran University of Medical Sciences, 1449614535 Tehran, Iran; 3 Department of Biochemistry, Faculty of Medicine, Iran University of Medical Sciences, Tehran, Iran; 4 Endocrinology and Metabolism Research Center, Endocrinology and Metabolism Clinical Sciences Institute, Tehran University of Medical Sciences, Tehran, Iran

**Keywords:** Permanent, Neonatal, Diabetes mellitus, Repaglinide, Case report

## Abstract

**Background:**

Permanent neonatal diabetes mellitus (PNDM) presents with dehydration and hyperglycemia, which usually occurs during the first 12 months of life. Activating mutations of beta-cell adenosine triphosphate-sensitive potassium [KATP] channel subunits that cause opening of the channel are associated with PNDM. Some patients with PNDM respond to administration of a sulfonylurea derivative, which has long action on blood glucose even during hypoglycemia and has an apoptotic effect on beta cells. However, there have been no reports regarding treatment with meglitinide (repaglinide), which has rapid and short duration of action during the rise in blood glucose after meals that is more similar to beta cell function. It has no effects during hypoglycemia, so it does not cause neurological damage, and has no apoptotic effect on beta cells. We report herein the effects of repaglinide administration in the management and clinical outcome of two patients with PNDM during 9 and 10 years of follow-up.

**Case presentation:**

Two Iranian infants were brought to our institution with poor general condition, dehydration, lethargy, and poor feeding. They had diabetic ketoacidosis at 52 days and 3.5 months of age, respectively. Their genetic analysis revealed mutations in the *KCNJ11* gene encoding KIR6.2, so they both had PNDM. After treatment of diabetic ketoacidosis with insulin, they responded to sulfonylurea (glibenclamide) treatment, but were switched to repaglinide because of blood sugar fluctuations in terms of hyper- and hypoglycemia. Repaglinide was administered with the dosage of 0.04 mg/kg/day divided before every meal.

**Results:**

The patients were 10 and 9 years old at the last visit, with normal growth parameters. The values of self-monitored blood glucose were well-controlled, and the hemoglobin A1C (HbA1C) levels ranged from 3.6 to 6.4% during the follow-up period. There was no complication of diabetes, neurological disorder, or adverse effects related to repaglinide.

**Conclusion:**

In every neonate or infant < 6 months of age with diabetes mellitus, PNDM should be considered. A trial of oral repaglinide can be performed and substituted for glibenclamide for prevention of hypoglycemia, neurological damage, and apoptosis of beta cells during long-term administration.

## Background

Diabetes is generally divided into different categories, including type 1 diabetes mellitus (DM), which is caused by autoimmune-mediated beta cell destruction; diabetes related to pancreatic injury; and type 2 diabetes mellitus, with insulin resistance as the main cause of the disease and with a genetic background [1, 2]. Genetic defects of beta cell function cause maturity-onset diabetes of the young (MODY), which has 10 types and is characterized by onset before 25 years of age, with an autosomal dominant pattern of inheritance and occurrence in three consecutive generations. Mutations in various genes including *HNF4A* and *GCK* are responsible for different types of MODY [3]. Mitochondrial gene defects cause maternally inherited diabetes and deafness—for example, Wolfram syndrome 1, which is characterized by DM, diabetes insipidus, deafness, and optic atrophy, and Wolfram syndrome 2, consisting of optic atrophy, DM, and deafness, but no diabetes insipidus [3].

Neonatal diabetes mellitus of a transient type presents during the first week of life and persists several weeks or months, with median duration of 12 weeks and spontaneous resolution. Permanent neonatal diabetes mellitus (PNDM) occurs in the first 12 months of life, with onset typically between birth and 6 months, and mean age of presentation of 5 weeks [3, 4]. Its incidence is reported as 1/90,000 to 1/500,000 live births [5, 6]. The most common causes of PNDM are (1) activating mutations in the *KCNJ11* gene which encodes Kir6.2, the pore-forming subunit of the ATP-sensitive potassium channel (K-ATP channel) and (2) the *ABCC8* gene which encodes the sulfonylurea receptor 1 (SUR1) subunit of the channel [7–10]. This disorder presents with diabetic ketoacidosis (DKA) or marked hyperglycemia due to low insulin levels and is usually treated with insulin [8]. However, there are reports of successful switching of treatment from insulin to sulfonylurea in these patients [5–9]. Some children with PNDM also have neurological problems. The combination of developmental delay, recurrent seizures (epilepsy), and neonatal diabetes is called DEND syndrome. The intermediate type of this syndrome is characterized by a milder developmental delay, without epilepsy [7]. The cause of this syndrome is not explained in the literature, but may be due to unnoticed hypoglycemic events during treatment with a sulfonylurea, which has a long duration of action even during fasting and hypoglycemia in an infant who cannot explain the hunger. On the other hand, sulfonylurea has an apoptotic effect on beta cells that may be due to long-acting stimulation of these cells that are not like normal physiology. So far, there has been no report of treatment with meglitinide (repaglinide), which has a rapid and short-acting function and has no effect during hypoglycemia, and its function is more similar to the physiology of normal beta cells and has no apoptotic effect on these cells. This is a report of two patients who were first treated with insulin then replied to sulfonylurea, and because of recurrent hyper- and hypoglycemia, were successfully treated with meglitinide (repaglinide) and their follow-up for 9 and 10 years. These patients have good metabolic control without hypoglycemia or neurological problems.

## Case presentations

### Case 1 (N.NE)

A 52-day-old Iranian girl was referred to us due to lethargy, fever, and poor feeding, which had started 5 days earlier. She was the first child of unrelated healthy parents. There was no family history of diabetes before 25 years of age in three generations. She was born on March 21, 2011, full term by cesarean section (CS), with birth weight of 2.600 kg and height of 48 cm. On physical examination, she was conscious but had drowsiness, dehydration, tachycardia, tachypnea, and sighing Kussmaul respiration. Her weight was 3.700 kg, with height of 52 cm (at the third percentile of the Centers for Disease Control and Prevention [CDC] 2000 clinical growth chart curves), respiratory rate 40 breaths/minute, pulse rate 197/minute, body temperature 38.2 °C, and blood pressure 97/49 mmHg.

Laboratory data showed severe DKA (Table 1). Fluid therapy and low-dose insulin infusion improved DKA, after which maintenance therapy with insulin was commenced. Eleven days later, we decreased the amount of insulin so that blood glucose was maintained between 200 and 300 mg/dL, and glibenclamide was administered to test its effect. It was effective in lowering blood glucose, so it was gradually substituted for insulin. Blood glucose was monitored before and 2 hours after every meal. She received glibenclamide until 2.4 years of age, but frequent monitoring of blood glucose showed some hypoglycemia before meals (minimum = 50 mg), while postprandial glucose was normal or high. Treatment was therefore switched to repaglinide (Novonorm, Novo Nordisk, Denmark), which is short-acting and is claimed to have no effect on insulin release at low blood glucose levels. The time course of treatment and follow-up is shown in Table 2.

**Table 1:** Laboratory tests at entry

Patients	BG mg/dL	Blood pH	HCO3 mEq/L	Serum Na* mEq/L	Serum K mEq/L	Urine ketone	BUN mg/dL	Cre mg/dL
Case 1	750	6.88	< 3	162	4.3	+2	11	0.4
Case 2	500	6.98	3.9	150.4	4.8	+2	26	1

*Corrected serum sodium

*BG* blood glucose, *BUN* blood urea nitrogen, *Cre* serum creatinine

**Table 2:** Age at appearance of signs and start of different treatments

Patients	Age	Age at appearance of signs and start of different treatments
Case # 1 N.NE	47 days	Signs appeared
	52 days	Came to hospital with DKA; treatment: low-dose insulin infusion, fluid therapy, then maintenance insulin therapy
	58 days	Glibenclamide
	2.4 years	Repaglinide
	10 years	Repaglinide; no adverse events
Case # 2 I.M.	3 months and 5 days	Signs appeared
	3 months and 15 days	Came to hospital with DKA; low-dose insulin infusion, fluid therapy, then maintenance insulin therapy
	3 months and 20 days	Glibenclamide
	4 months and 20 days	Repaglinide
	9 years	Repaglinide; no adverse events

*DKA* diabetic ketoacidosis

Because one tablet of repaglinide is 0.5 mg, compared to 5 mg glibenclamide, and the dosage of glibenclamide is 0.4 mg/kg/day [1], repaglinide dosage was calculated according to its potency as 0.04 mg/kg/day divided into three or more doses before each meal based on the patient’s age and frequency of meals. Preparations of the medicine were made by dissolving one tablet in 10 ml of water and calculating the dosage by volume of solution in every administration and spill-out of the extra. Adjustment of the dosage with weight was done during the follow-up period, which was effective for normal glucose maintenance.

Genetic analysis was done in the molecular genetics laboratory of the Medical School of the University of Exeter in the UK. Sequence analysis of the *ABCC8*, *KCNJ11*, and *INS* genes was performed. Analysis of coding and flanking intronic regions of the *KCNJ11*, *INS,* and *ABCC8* genes (NM_000525.3, NM_000207.2, NM_001287174.1) was done by Sanger sequencing. A heterozygous missense mutation was identified, with details as follows: gene: *KCNJ11*; location: exon 1; DNA description: c.602G>A; protein description: p.Arg201His (p.R201H). Therefore, a diagnosis was PNDM was made.

At the last visit, she was 10 years old, with height of 143 cm (75th percentile) and weight of 50 kg (97% of the CDC 2000 curves). Hemoglobin A1C (HbA1C) was measured every 3 months during the follow-up period, with mean ± SD of all of the measurements calculated as 4.6 ± 0.7 (range 3.6–6.4). The blood glucose level from 59 recent self-monitored measurements was 123.36 ± 18 mg/dL (90–173). Neurodevelopmental assays were normal by both physical examination and questions of the parents. Ophthalmic examination was done and found to be normal.

### Case 2 (I.M.)

A 3.5-month-old Iranian girl was referred due to vomiting, respiratory distress, fever, and lethargy. She had some of the signs since 10 days before presentation. She was the second child of a family with two children, and the other sibling was healthy. Both parents were healthy and first cousins. There was no family history of diabetes before 25 years of age in three generations. She was born on May 12, 2012, full term by elective CS, with birth weight of 2.500 kg. At the time of admission, she was in a light coma and dehydrated. Her weight was 5 kg (10th percentile) and height 53 cm (under the fifth percentile). Respiratory rate was 50, and pulse rate 170, with normal blood pressure and temperature. She had severe DKA (Table 1). DKA improved after 26 hours of fluid therapy and low-dose insulin infusion. Glibenclamide was substituted for insulin after 5 days, with a dosage of 0.4 mg/kg once daily. The treatment was changed to repaglinide 1 month later because some hypoglycemia (minimum = 52 mg/dL) occurred, in an effort to keep 2-hour postprandial blood glucose at normal levels. The daily dosage was 0.04 mg/kg, and the method of preparation was the same as in the first case. It was divided into eight separate doses before each milk feeding, and when she became older, the daily dose was divided before every meal. During the last visit, she was 9 years old, with weight of 22.5 kg and height of 122 cm. The time course of treatment and follow-up is shown in Table 2.

Weight and height were always in the third percentile of the CDC 2000 curves during follow-up. HbA1C was measured every 3 months, with a mean ± SD of 4.7 ± 0.5 (4.2–6.3). The blood glucose level from 93 recent self-monitored measurements was 118.7 ± 16.4 mg/dL (88–167). Genetic analysis was done in the genes mentioned above and also on the *EIF2AK3* gene in the same center. The result was the same as in the first case. The patient showed normal neurological and ophthalmological examination results.

### Diabetic complications

Routine physical examination and laboratory tests for diabetic care were done for both patients in different periods: Measurement of HbA1C and physical examination was done every 3 months; lipid profile, biochemical profile, thyroid, liver, and renal function tests were assayed every year. There were no diabetic complications in any organ of the body in either patient, and they had normal neurodevelopment and body growth during 9 and 10 years of follow-up. Annual ophthalmic examination results were also normal, with no retinopathy. Tolerability to the medicine has been very good, with no adverse effects.

## Discussion

Switching to a sulfonylurea derivative (glibenclamide) in PNDM has been reported in different studies in patients with both *KCNJ11* and *ABCC8* genetic mutations [5–8, 11]. However, treatment with repaglinide, which is from the meglitinide group, has not been previously reported in this disorder. Here, we present two cases of patients with PNDM who were successfully treated with repaglinide. In both cases, missing a dose resulted in increased blood glucose to 200 mg/dL, proving the permanent nature of the disease and the transient effect of repaglinide. Neonatal onset of disease and its permanent nature together with the lack of anomalies such as deafness and optic atrophy ruled out other etiologies of diabetes, including autoimmunity and various types of MODY. The results of genetic analysis further confirmed the diagnosis. Long-term follow-up showed good management of the condition with repaglinide in both cases.

Repaglinide is a carbamoylmethyl benzoic acid derivative that inhibits opening of the ATP-sensitive potassium channel and increases insulin release in a glucose-dependent manner. Its mechanism of action is similar to that of sulfonylureas, but it has some differences and more advantages. It acts on a different binding site and has a weaker binding affinity and faster dissociation from the SUR1 subunit binding site. It does not directly promote insulin exocytosis. In contrast to a sulfonylurea, which is an insulin secretagogue with long duration, repaglinide function is rapid and short-acting and has no effect on insulin release if the glucose level is low; it has little effect on insulin levels overnight, between meals, or during hypoglycemia [12], and it reduces postprandial blood glucose levels [12]. Thus it has a more physiological action, with low risk of causing hypoglycemia [13]. Some individuals with PNDM have DEND syndrome. In a large cohort of PNDM patients carrying *KCNJ11* mutations in long-term follow-up (17), among 81 patients, 38 (64%) had CNS problems. Although some initial improvement in CNS features was seen in 18 (47%) of 38 patients after switching from insulin to sulfonylureas, this effect was usually incomplete and subsequently plateaued. These findings may be explained by unnoticed hypoglycemic events during treatment with sulfonylureas and insulin, especially in infants and during overnight periods.

Repaglinide is rapidly absorbed after oral administration and appears in the circulation after 15 minutes, with a peak level within 30–60 minutes that coincides with the rise in blood glucose after a meal. It is also rapidly metabolized and has an elimination half-life of approximately 1 hour. Repaglinide binds to proteins in plasma, and its absolute bioavailability is approximately 62.5% [13]. While single-dose studies with glibenclamide tablets in normal subjects demonstrated significant absorption within 1 hour, peak drug levels were seen at about 4 hours, and low but detectable levels at 24 hours, with hypoglycemia representing a significant adverse event, while repaglinide has no effect on insulin secretion when blood sugar is not high [10, 11]. In an experimental *in vitro* study on beta cells from donors, it was shown that repaglinide has less apoptotic effect on beta cells compared to glibenclamide [14]. In our patients, repaglinide was very effective in maintaining a normal range of blood glucose without any hypoglycemia and showed no side effects during 9 and 10 years of follow-up in two patients. The patients had normal growth and development, normal HbA1C levels were maintained, and they had no diabetic complications or neurological damage during the follow-up period.

Patients with HNF1A and HNF4A MODY (MODY3) also show sensitivity to sulfonylurea. Clinical features similar to HNF1A/4A MODY can also be caused by mutations in the *ABCC8* gene; however, this disorder is different from PNDM, in that MODY3 generally presents later in life [15]. The early presentation of disease together with genetic analysis ruled out this type of disease in our patients.

## Conclusions

Repaglinide can be administered for the treatment of patients with PNDM. We observed no complications of diabetes or any neurological damage due to hypoglycemia and no adverse effects of the medicine in our patients.

## Data Availability

Data will be made available upon reasonable request.
